# Metal Ion Concentrations in Body Fluids after Implantation of Hip Replacements with Metal-on-Metal Bearing – Systematic Review of Clinical and Epidemiological Studies

**DOI:** 10.1371/journal.pone.0070359

**Published:** 2013-08-07

**Authors:** Albrecht Hartmann, Franziska Hannemann, Jörg Lützner, Andreas Seidler, Hans Drexler, Klaus-Peter Günther, Jochen Schmitt

**Affiliations:** 1 University Hospital Carl Gustav Carus Dresden, University Centre for Orthopaedics and Trauma Surgery, Dresden, Germany; 2 University Hospital Carl Gustav Carus Dresden, Centre for Evidence-Based Health Care, Dresden, Germany; 3 Dresden University of Technology, Occupational and Social Medicine, Dresden, Germany; 4 University Erlangen-Nuremberg, Occupational, Social and Environmental Medicine, Erlangen, Germany; University of Michigan, United States of America

## Abstract

**Introduction:**

The use of metal-on-metal (MoM) total hip arthroplasty (THA) increased in the last decades. A release of metal products (i.e. particles, ions, metallo-organic compounds) in these implants may cause local and/or systemic adverse reactions. Metal ion concentrations in body fluids are surrogate measures of metal exposure.

**Objective:**

To systematically summarize and critically appraise published studies concerning metal ion concentrations after MoM THA.

**Methods:**

Systematic review of clinical trials (RCTs) and epidemiological studies with assessment of metal ion levels (cobalt, chromium, titanium, nickel, molybdenum) in body fluids after implantation of metalliferous hip replacements. Systematic search in PubMed and Embase in January 2012 supplemented by hand search. Standardized abstraction of pre- and postoperative metal ion concentrations stratified by type of bearing (primary explanatory factor), patient characteristics as well as study quality characteristics (secondary explanatory factors).

**Results:**

Overall, 104 studies (11 RCTs, 93 epidemiological studies) totaling 9.957 patients with measurement of metal ions in body fluids were identified and analyzed. Consistently, median metal ion concentrations were persistently elevated after implantation of MoM-bearings in all investigated mediums (whole blood, serum, plasma, erythrocytes, urine) irrespective of patient characteristics and study characteristics. In several studies very high serum cobalt concentrations above 50 µg/L were measured (detection limit typically 0.3 µg/L). Highest metal ion concentrations were observed after treatment with stemmed large-head MoM-implants and hip resurfacing arthroplasty.

**Discussion:**

Due to the risk of local and systemic accumulation of metallic products after treatment with MoM-bearing, risk and benefits should be carefully balanced preoperatively. The authors support a proposed „time out“ for stemmed large-head MoM-THA and recommend a restricted indication for hip resurfacing arthroplasty. Patients with implanted MoM-bearing should receive regular and standardized monitoring of metal ion concentrations. Further research is indicated especially with regard to potential systemic reactions due to accumulation of metal products.

## Introduction

Total hip arthroplasty (THA) for patients with osteoarthritis is one of the most successful surgical interventions in general inducing substantial improvement of health-related quality of life of affected patients [1]. Aseptic loosening is a typical long-term complication that significantly determines implant survival. Compared to regular bearings with conventional polyethylene, one advantage of metal-on-metal (MoM) bearings is that they produce less volumetric wear [2]. However, MoM hip replacements may release metallic products (i.e. particles, ions, metallo-organic compounds) due to wear and corrosion [3], [4]. Metal ions from the corresponding alloying element (i.e. cobalt – Co, chromium – Cr, titanium – Ti, nickel – Ni, molybdenum – Mo) can be measured in the joint itself as well as in surrounding tissue and body fluids, and may potentially cause local and or/systemic adverse reactions [5], [6], [7], [8]. Recently, metal-related local adverse reactions (i.e. adverse reactions to metal debris, ARMD) in patients with MoM hip replacement gained substantial scientific and public attention [9], [10], [11], [12], [13], [14]. Endoprothesis registries from Australia and the UK [15], [16], [17] indicated increased revision rates following hip replacements with MoM-bearing – especially large-head THA and hip resurfacing arthroplasty (HRA). The risk of local adverse reactions of MoM-THA has been reported to correlate with the level of systemic metal ion concentrations [5], [6], [8], [13], [18], [19], [20]. However, several clinically relevant issues related to the safety of MoM-THA such as the impact of the different types of MoM implants on metal ion concentrations and related risks and the long-term course of postoperative metal ion concentrations require further investigation. Case reports [21], [22] also suggest systemic risks due to metal burden after MoM-THA, but systematic research on this important issue is missing. There is substantial evidence from occupational medicine indicating that employees in metal producing and -processing plants exposured to chromium(VI) resp. cobalt compounds are at increased risk of nasal septum ulcerations, lung cancer resp. and cardiomyopathy [23], [24], [25]. It has to be noted, however, that toxicity of Cr(III) compounds is substantially lower than those of Cr(VI) compounds. Because the primary route of metal exposure in occupational medicine is the respiratory tract, generalizability of these findings to patients with metallic hip replacement remains unclear.

Despite the current uncertainty a systematic review on the safety of MoM-hip replacements is missing. We systematically appraised published clinical and epidemiologic studies to clarify the following issues related to the safety of MoM-hip replacement:

- What are median and maximum metal ion concentrations following MoM-hip replacement?
- Which patient and implant related risk factors exist for elevated metal ion concentrations following MoM-hip replacement?
- To what extent does the metal ion concentration after MoM-hip replacement predict local and systemic adverse reactions?

## Methods

We undertook a systematic review to identify, summarize, and critically appraise the clinical and epidemiological evidence concerning the impact of metalliferous hip replacements on metal ion levels in body fluids. In addition to the type of bearing as the hypothesized determinant of metal ion concentrations we were particularly interested in patient characteristics as well as study quality characteristics as potential secondary determinants of metal ion concentrations after THR, and in the clinical consequences resulting from increased metal ion concentrations.

### Inclusion criteria

All randomized controlled trials (RCTs) and epidemiological studies (cohort, case-control and cross-sectional studies, case series) with metal ion measurement (cobalt, chromium, titanium, nickel, molybdenum) in body fluids (full blood, serum, plasma, erythrocytes, synovia, urine) after implantation of metalliferous hip replacements in at least 20 patients were considered eligible. Studies were required to be published as an original article in English, German, or French language to be included.

### Literature search

Systematic electronic literature searches were conducted in PubMed and EMBASE (until January 19, 2012). Combinations of MeSH-terms were used to identify relevant trials with a high sensitivity. The exact search string used is provided in table 1. Systematic electronic search was supplemented by hand search in the reference lists of the papers included, as well as all articles published in the „Journal of Bone and Joint Surgery British“ between 2007 and 2011.Screening of titles and abstracts as well as full-text articles was done independently by two reviewers (F.H., A.H.). Disagreements were resolved by discussing within the whole team of reviewers.

**Table 1:** Search string.

#	Suchstring
**1**	“Arthroplasty, Replacement, Hip”[Mesh] OR “Hip Prosthesis”[Mesh]
**2**	total hip arthroplast*[All Fields] OR “THA”[All Fields] OR hip arthroplast*[All Fields] OR total hip replacement*[All Fields] OR hip replacement*[All Fields] OR “hip prosthesis”[All Fields]
**3**	“surface replacement”[All Fields] OR “hip resurfacing”[All Fields] OR hip resurfacing arthroplast*[All Fields] OR “HRA”[All Fields] OR surface replacement arthroplast*[All Fields] OR “articular surface replacement”[All Fields] OR “ASR”[All Fields] OR surface arthroplast*[All Fields] OR “Birmingham Hip Resurfacing”[All Fields] OR “BHR”[All Fields]
**4**	#1 OR #2 OR #3
**5**	“Chromium”[Mesh] OR “Chromium Alloys”[Mesh] OR “Cobalt”[Mesh] OR “Molybdenum”[Mesh] OR “Titanium”[Mesh] OR “Nickel”[Mesh]
**6**	“Chromium”[All Fields] OR “Cr”[All Fields] OR Titanium*[All Fields] OR “Ti”[All Fields] OR Nickel*[All Fields] OR Cobalt*[All Fields] OR “Co”[All Fields] OR Molybdenum*[All Fields] OR „Mo“[All Fields]
**7**	#5 OR #6
**8**	“Blood”[Mesh] OR “Urine”[Mesh] OR “Tissues”[Mesh]
**9**	“blood”[All Fields] OR “serum”[All Fields] OR “plasma”[All Fields] OR “urine”[All Fields] OR “tissue [All Fields] OR “tissues”[All Fields]
**10**	#8 OR #9
**11**	#4 AND #7 AND #10
**12**	#11 NOT (letter[pt] OR editorial[pt] OR comment[pt] OR review[pt] OR meta-analysis[pt])
**13**	#12 NOT ((animals[Mesh:NoExp]) NOT (humans[Mesh]))
**14**	#12 NOT ((animals[Mesh:NoExp]) NOT (humans[Mesh])) Limits: only items with abstracts

### Data abstraction

The following information was abstracted from the studies included using standardized and beta tested evidence tables:

- study characteristics (e.g. author, geographical region, study design, time points of assessment, follow-up period).
- Patient characteristics (e.g. number of patients included and followed up to each point of assessment of metal ion level, UCLA – University of California Los Angeles score [26], body mass index).
- Implant characteristics (type of bearing material and head size in three different groups of implants: small head (SH-)THA with head diameter ≤32 mm, stemmed large-head (LH-)THA with head diameter ≥36 mm, HRA).
- Implant position (inclination of acetabular cup in the frontal plane).
- Details on metal ion assessment, i.e. type of metal ions assessed (cobalt, chromium, titanium, nickel, molybdenum), the medium of assessment, method of analysis, metal ion levels (median, mean, interquartile range (IQR), outliers, definition of outliers.
- Clinical results, i.e. local adverse reactions such as ARMD – „adverse reactions to metal debris”, systemic adverse reactions.

### Rating of methodological study quality

Standardized study quality assessment was based on the CASP und SIGN Checklists [27], [28]. Based on consented and a priori defined study quality criteria the risk of bias was rated for each study with the following categories:

- very low risk of bias: „++“
- low risk of bias: „+“
- high risk of bias: „−“

We considered the following criteria to increase the risk of bias:

- no consideration of confounding and/or explanatory factors such as other metallic implants, type, size, and position of implant.
- missing information on the methods used to measure metal ion levels.
- missing reference group without THR.
- missing preoperative (baseline) metal ion assessment.
- missing outcome data, i.e. loss to follow-up >10% [29].

### Evidence-synthesis

The qualitative evidence-synthesis included a comparison of the pre- vs. postoperative metal ion concentrations (median (alternatively: mean), IQR, maximum values) stratified by type of bearing as the primary explanatory factor. Patient characteristics (mean age, sex ratio) and implant characteristics (bearing size and position) were considered as secondary explanatory factors. Additionally, the definition of cut-off levels of metal ion concentrations in different studies was compared.

RCTs and epidemiological studies were analyzed separately. The detection limit of metal ions depends on the method and device used. The range of the detection limits reported and the handling of values below the detection rate was also part of the qualitative synthesis of the published evidence.

The course of metal ions in body fluids over time was assessed based on studies reporting baseline serum Co-values and at least 2 postoperative Co-measurements. In these studies, we investigated the course of median serum Co-concentration after implantation of hip replacements with different kinds of metal-on-metal bearings.

Metal ion concentrations in all tables and figures below generally relate to patients with unilateral THR, unless stated differently. We initially planned to conduct a quantitative summary (meta-analysis) of the results of qualitatively homogeneous studies with very low or low risk of bias.

Data on all relevant metal ions (cobalt, chromium, titanium, nickel, molybdenum) was extracted from the studies included. In the results section, we present cobalt levels as the proposed reference metal ion concentration after THR, as suggested by an international, multiprofessional expert panel [30].

## Results

**Figure 1:**
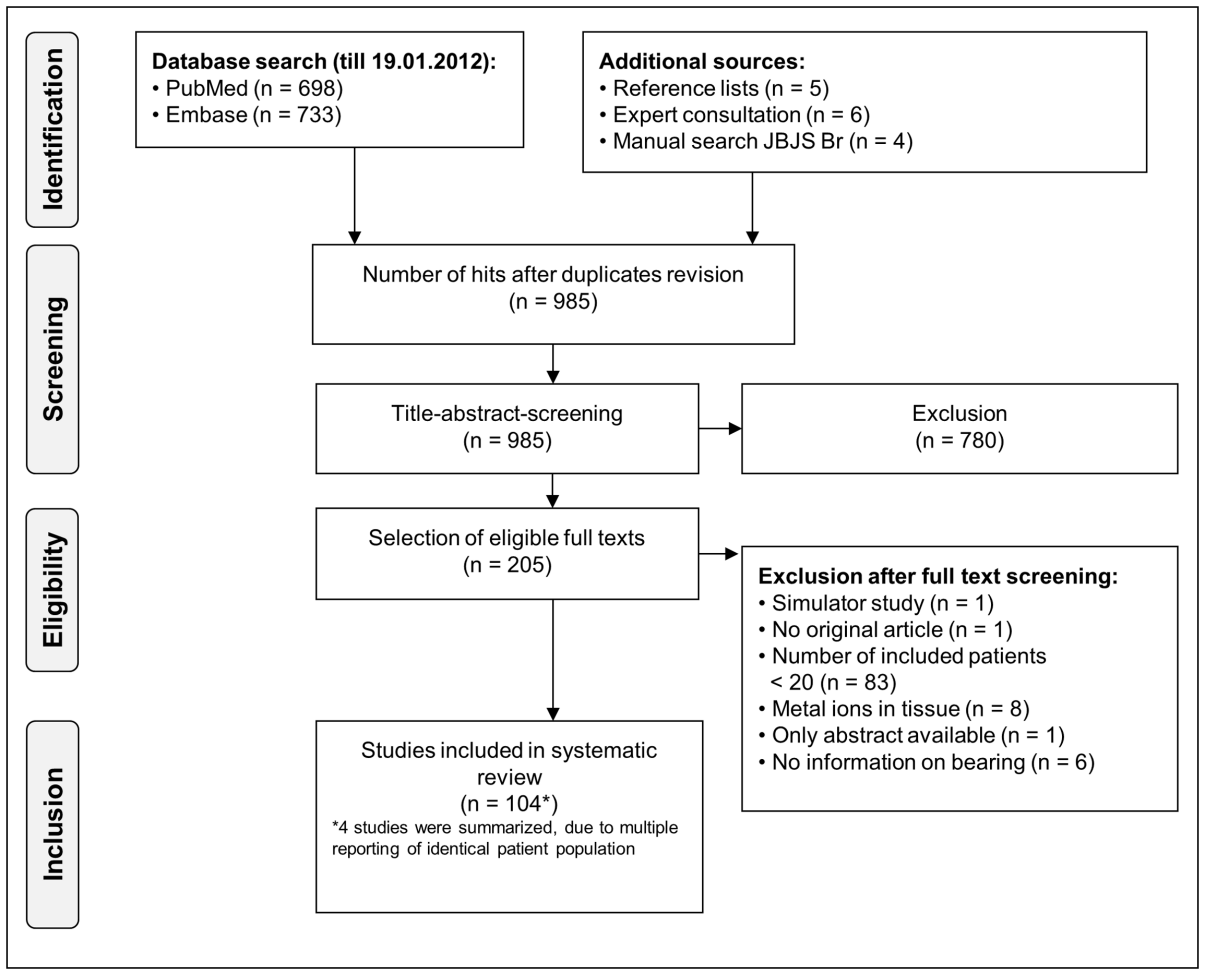
Study flow chart of in- and excluded studies. Figure 1 summarizes the yield of systematic search and study selection [31]. Overall, 104 studies (11 RCTs, 14 cohort studies, 1 case-control study, 55 cross-sectional studies, 2 case series) were identified and analyzed.

Figure 1 summarizes the yield of systematic search and study selection [31]. Overall, 104 studies (11 RCTs, 14 cohort studies, 1 case-control study, 55 cross-sectional studies, 23 case series) totaling 9.957 patients with measurement of metal ions in body fluids were identified and analyzed. The majority of studies were performed in Europe (n = 71) and North America (n = 26).

### Study characteristics RCTs

Table 1 summarizes the characteristics of the 11 RCTs included. RCTs were generally small and included between 13 and 117 patients. Two RCTs examined MoM vs. ceramic-polyethylene (CoP) [32], [33], five studies MoM vs. metal-polyethylene (MoP) [34], [35], [36], [37], [38], [39], two studies examined MoM vs. ceramic-ceramic (CoC) [26], [40], [41], and two MoM vs. hip resurfacing arthroplasty (HRA) [42], [43], [44]. LH-THA was investigated in one US-American study [35]. All other RCTs investigated MoM SH-THA.

Despite some methodological limitations such as missing description of patient recruitment/randomization, incomplete information on patient characteristics, and incomplete outcome data, all RCTs were considered as having low risk of bias (Table 2).

**Table 2:** Study characteristics of included randomized controlled trials (RCTs).

Reference							Intervention		Bearing				Head size			Manufacturer							
													THA										
	Study design	Geographical region	Number of patients^1^	Age (mean)^2^	Proportion women (%)	BMI^3^	THA^4^ (%)	HRA^5^ (%)	MoM	MoP	CoC	CoP	Small^6^	Large^7^	Both	Zimmer**	DePuy***	Wright Medical Technology	Others	Inclination	Anteversion	UCLA Activity Score	Study quality
Brodner 1997	RCT	Austria	27/55	59/60*	66/69		100		•			•	•			•							+
Brodner 2003	RCT	Austria	50/100	58/60*	56/70	•	100		•			•	•			•			•	•		•	+
Dahlstrand 2009	RCT	Sweden	54	65	50	•	100		•	•			•			•							+
Engh 2009	RCT	USA	91				100		•	•					•		•			•			+
Grübl 2006	RCT	Austria	13/28	67/62	77/64	•	100		•		•		•			•			•	•		•	+
Hailer 2011	RCT	Sweden	85			•	100		•	•			•			•							+
MacDonald 2003	RCT	Canada	41				100		•	•			•						•				+
Smolders 2011b	RCT	Netherlands	71		41	•	46	54	•				•			•		•				•	+
Vendittoli 2007+2010	RCT	Canada	117	50*	36	•	45	55	•				•			•				•		•	+
Weissinger 2011	RCT	Austria	42/80	66	67/68	•	100		•		•		•			•			•	•		•	+
Zijlstra 2009+2010	RCT	Netherlands	43				100		•	•			•						•				+

Gathered information refer to patients with at least one metallic part of the bearing being investigated on metal ions.

Studies were summarized due to multiple reporting of identical patient population.

RCT: randomised controlled trial; comparison of 2 interventions, being different regarding e.g. bearing or type of implant; incl. metal ion measurement for all groups.1: Number of patients (with at least one metallic part of the bearing) being investigated on metal ions/total number of patients being investigated.

on metal ions.

2: Rounded mean age of patients with at least one metallic part of the bearing/rounded mean age of total number of patients being investigated.

on metal ions.

* : Rounded weighted mean.

3: BMI  =  Body Mass Index.

4: THA  =  Total hip replacement.

5: HRA  =  Hip resurfacing arthroplasty.

MoM: Metal-on-Metal bearing.

MoP: Metal-on-Polyethylene bearing.

MoC: Metall-on-Ceramic bearing.

CoC: Ceramic-on-Ceramic bearing.

CoP: Ceramic-on-Polyethylene bearing.

6: Femoral head size 28–32 mm; small head.

7: Femoral head size ≥36 mm; large head.

** incl. Protek, AlloPro, Sulzer Orthop., Centerpulse, Zimmer.

*** incl. Landander.

UCLA: Activity Score, University of California, Los Angeles.

### Determination of metal ions in RCTs

Table 3 presents details of metal ion assessment in the RCTs included. All RCTs reported pre- and postoperative Co-concentrations. Nine [32], [33], [34], [35], [36], [38], [39], [40], [41], [43], [44] RCTs preferred serum as medium for metal ion assessment. Long-term measurement ≥24 months were reported in five RCTs.[33], [36], [37], [38], [39], [41].

**Table 3:** Points of time, investigated mediums, methods of analysis, and reporting of results of metal ion measurement in included RCTs.

Reference	Metal ion measurement^1^					Metal ions		Medium				Technique		Outcome reported
	Preoperative	≤6 Months	>6–≤12 Months	> 12–≤24 Months	> 24 Months	Co	Cr	Whole blood	Erythrocytes	Serum	Urine^2^	AAS^3^	ICP-MS^4^	
Brodner 1997	•	•	•			•				•		•		mean, median, IQR
Brodner 2003	•	•	•	•	•	•				•		•		median, IQR
Dahlstrand 2009	•	•	•	•		•	•			•			•	mean, median, IQR
Engh 2009	•	•	•	•		•	•		•	•	•		•	median, IQR
Grübl 2006	•		•			•				•		•		median, IQR, range
Hailer 2011	•				•	•	•			•			•	mean
MacDonald 2003	•	•	•	•	•	•	•		•		•		•	median, IQR
Smolders 2011b	•	•	•	•		•	•	•					•	median, range
Vendittoli 2007+2010	•	•	•	•		•	•	•	•	•			•	mean, SD, range, median, IQR
Weissinger 2011	•		•		•	•				•		•		mean, median, IQR
Zijlstra 2009+2010	•			•	•	•	•			•		•		median, range

Studies were summarized due to multiple reporting of identical patient population.

2: Incl. all kinds of metal ion measurement in urin, e.g. 12- or 24 h urine.

3: AAS  =  All procedures of atomic absorption spectrometry.

4: ICP-MS  =  all procedures of inductively coupled plasma mass spectrometry.

n. r.  =  Not reported.

SD: Standard deviation; SEM: Standard error of the mean; IQR: Interquartile range.Metal ion concentration in RCTs.

As detailed in table 4, median Co-concentrations were elevated at each postoperative assessment after implantation of MoM SH-THA, MoM LH-THA, and HRA compared to THA with only one or without metallic part of the bearing. Following MoM SH-THA median serum Co-concentrations varied between 0.66–1.0 µg/L at 6 months [32], [33], [34], [35] and 0.73–1.2 µg/L at 2 years [33], [34], [35], [41] after surgery. After MoM LH-THA median serum Co-levels of 0.66 µg/l and 0.73 µg/L were reported at 6-month and 2-year follow-up, respectively [35]. After HRA, median whole blood Co-concentrations varied between 0.78 and 1.3 µg/L up six months und between 0.16 and 1.2 µg/L two years postoperatively [42], [43], [44]. The only direct comparison between MoM SH- and LH-THA revealed no relevant differences in the median serum Co-concentration [35].

**Table 4:** Concentrations of Co in µg/L for several interventions in RCTs.

	Cobalt µg/L – Comparison (Median; IQR; n)							
Reference	Comparison	Medium	Preoperative	6 Months (±3)	12 Months (±3)	24 Months (±6)	48 Months (±6)	120 Months
Brodner 1997	CoP SH-THA	Serum	0.15; 0.15–0.15; 28	0.15; 0.15–0.15; 28	0.15; 0.15–0.15; 28			
Brodner 2003	CoP SH-THA	Serum	0.15; 0.15–0.15; 50	0.15; 0.15–0.15; 50	0.15; 0.15–0.15; 50	0.15; 0.15–0.15; 50	0.15; 0.15–0.15; 37	
Dahlstrand 2009	MoP SH-THA	Serum	0.1; 0.05–0.25; 26	0.1; 0.05–0.2; 26	0.15; 0.06–0.3; 26	0.25; 0.1–0.4; 26		
Engh 2009	MoP SH-THA	Serum	0.15; 0.10–0.22; 34	0.15; 0.11–0.20; 30	0.12; 0.10–0.17; 31	0.14; 0.09–0.19; 28		
Grübl 2006	CoC SH-THA	Serum	0.15; 0.15–0.15; 15		0.40; 0.15–0.70; 15****			
Hailer 2011	MoP SH-THA	Serum	0.16; 44**				0.24; 17** ^2^	
MacDonald 2003	MoP SH-THA	Erythrocytes	0.11; 0.09–0.15; 18	not reported	not readable	0.17; 0.12–0.23; 18^3^		
Smolders 2011b	MoM SH-THA	Whole blood	0.1; 0.1–0.6; 29****	0.85; 0.1–4.0; 29****	1.0; 0.1–4.2; 28****	0.9; 0.1–2.7; 17****		
Vendittoli 2007+2010	MoM SH-THA	Whole blood	0.15; 0.06–0.42; 36***	0.87; 0.25–3.57; 33***	0.81; 0.23–2.10; 31***	0.94; 0.24–4.89; 24***		
Weissinger 2011	CoC SH-THA	Serum	0.15; 38*		not reported	0.15; 0.15–0.4^4^		
Zijlstra 2009+2010	MoP SH-THA	Serum	0.24; 0.18–0.65; 19****			0.18; 0.18–1.06; 19****	0.30; 0.29–1.65; 14****5	0.50; 0.40–1.30; 13****6

	Cobalt µg/L – Intervention (Median; IQR; n)							
Reference	Intervention	Medium	Preoperative	6 Months (±3)	12 Months (±3)	24 Months (±6)	48 Months (±6)	120 Months
Brodner 1997	MoM SH-THA	Serum	0.15; 0.15–0.15; 27	1.0; 0.35–1.35;27	1.1; 0.65–2.6; 27			
Brodner 2003	MoM SH-THA	Serum	0.15; 0.15–0.15; 50	1.0; 0.5–1.4; 50	1.0; 0.6–2.2; 50	0.75; 0.4–2.85; 50	0.9; 0.4–1.7;36	
Dahlstrand 2009	MoM SH-THA	Serum	0.05; 0.05–0.1; 28	0.75; 0.5–1.05; 28	0.8; 0.5–1.05; 28	0.85; 0.65–1.3; 28		
Engh 2009	MoM SH-THA	Serum	0.16; 0.09–0.29; 24	0.72; 0.49–0.98; 21	0.72; 0.48–1.02; 22	0.77; 0.48–1.19; 21		
	MoM LH-THA	Serum	0.14; 0.08–0.22; 31	0.66; 0.37–0.85; 29	0.70; 0.49–1.10; 26	0.73; 0.47–1.99; 26		
Grübl 2006	MoM SH-THA	Serum	0.15; 0.15–0.15; 13		1.4; 0.5–10.5; 13****			
Hailer 2011	MoM SH-THA	Serum	0.09; 41**				0.86; 19** ^2^	
MacDonald 2003	MoM SH-THA	Erythrocytes	0.14; 0.09–0.17; 23	not reported	not readable	1.10; 0.66–2.43; 223		
Smolders 2011b	HRA	Whole blood	0.1; 0.1–0.8; 33****	1.3; 0.1–23; 33****	1.25; 0.6–8.3; 33****	1.2; 0.5–22; 16****		
Vendittoli 2007+2010	HRA	Whole blood	0.16; 0.06–1.05; 44***	0.78; 0.25–1.61 51***	0.67; 0.23–2.09; 57***	0.16; 0.20–2.89; 48***		
Weissinger 2011	MoM SH-THA	Serum	0.15; 42*		not reported	1.2; 0.8–2.74		
Zijlstra 2009+2010	MoM SH-THA	Serum	0.18; 0.18–1.77; 24****			0.77; 0.18–15.57; 24****	0.88; 0.29–7.02; 19**** 5	1.10; 0.50–11.0; 17**** 6

THA  =  Total hip arthroplasty; HRA  =  Hip resurfacing arthroplasty.

SH  =  Small head; LH  =  Large head; IQR  =  Interquartil range.

Hint:.

Values of the given studies were reported, if they fitted to the fixeds timeframes; if several measurements fitted to one timeframe, those values were reported.

being nearest to the timeframes given above; e. g. values were reported 3 months and 5 months postoperative > values 5 months postoperatively are shown in this table.

* Median, n.

** Mean, n.

*** Mean, range, n.

**** Median, range, n.

2 metal ion measurement mean 6.8 years postoperatively.

3 metal ion measurement mean 3.2 years postoperatively.

4 metal ion measurement median 24.3 months postoperatively.

5 metal ion measurement mean 67 months postoperatively.

6 metal ion measurement mean 121 months postoperatively.

Two RCTs directly compared MoM SH-THA vs. HRA and revealed qualitatively different results: While Smolders et al. [42] observed higher median and maximum Co-concentrations following HRA compared to MoM SH-THA, Vendittoli et al.[43], [44] did not observe relevant differences in mean Co-concentrations following HRA and MoM SH-THA. THA with only one metallic part of the bearing resulted in not or only slightly elevated serum Co-concentrations (Table 4).

### Study characteristics epidemiological studies

Table 5 summarizes aggregated characteristics of the epidemiological studies included. Table 6 provides details of the characteristics of each epidemiological study included. Metal ion concentrations following HRA were reported in 48 studies (52%). Information on inclination and anteversion were reported in 30 studies (32%) and in 16 (17%) studies, respectively. Information on mean age was reported in 66 studies (71%). The sex distribution of patients was reported in 64 studies (69%) and varied substantially with 19 to 90% of the study populations being female.

**Table 5:** Summary of study characteristics of included epidemiological studies.

**Study design**	Cohort study	N = 14
	Case-control study	N = 1
	Cross-sectional study	n = 55
	Case series	n = 23
**Geographical region**	Europe	n = 63
	North America	n = 23
	Asia	n = 6
	Australia	n = 1
**Patients characteristics**	number of patients (range)	20–789
	mean age (n = 70 studies)	37–70
	distribution of sex (n = 68 studies)	19–90% female
	**BMI** * **(n = 29 studies)**	
	**mean** ** (range of means, n = 25 studies)	22.8–28.5
	range of extreme values (n = 11 studies)	17–56.6
	**median** (range of medians, n = 9 studies)	26–28
	range of extreme values (n = 6 studies)	19–42

Implant characteristics		
**Intervention**	**THA**	**n = 70**
	MoM LH-THA	n = 26
	MoM SH-THA	n = 42
	MoP	n = 17
	MoC	n = 1
	CoC	n = 4
	CoP	n = 2
	**HRA**	**n = 47**
**Manufacturer**	Zimmer	n = 56
	DePuy	n = 26
	Smith&Nephew	n = 34
	Corin Group	n = 12
	Wright Medical Technology	n = 12
	others	n = 27
	not reported	n = 3
**Study quality**	++ (very low risk of bias)	n = 0
	+ (low risk of bias)	n = 7
	- (high risk of bias)	n = 86

* Including both sexes and different points of measurement (e.g. preoperative, or at follow up).

** Including weighted means for several study cohorts.

**Table 6:** Study characteristics of included epidemiological studies.

Reference	Study design	Geographical region	Number of patients^1^	Age (mean)^2^	Proportion women (%)	BMI^3^	Intervention		Bearing					Head size			Manufacturer						Inclination	Anteversion	UCLA Activity Score	Study quality
														THA												
							THA^4^ (%)	HRA^5^ (%)	MoM	MoP	MoC	CoC	CoP	Small^6^	Large^7^	Both	Zimmer**	DePuy***	Smith&Nephew****	Corin Group	Wright Medical Technology	Others				
Antoniou 2008	CO	Canada	174	58*	43		60	40	•	•						•	•	•							•	−
Beaulé 2011	CO	Canada	52	57*	25	•	50	50	•						•						•		•		•	+
Bernstein 2011a	CO	Canada	104	61	48		100		•							•	•	•					•	•	•	+
Boyer 2009	CO	France	109	54	49		100		•					•			•					•	•			+
De Souza 2010	CO	Great Britain	56	52	45			100	•											•						+
Garbuz 2010	CO	Canada	26/104	n. r./52*		•	50	50	•						•		•								•	−
Isaac 2009a	CO	Great Britain	60	54	65		100		•		•			•				•					•	•		+
Jacobs 1998	CO	USA	55	61*	53		100			•				•			•	•								+
Lavigne 2011	CO	Canada	137	54*	38	•	100		•						•		•	•	•			•	•		•	−
Lazennec 2009	CO	France	109	54	49	•	100		•					•			•					•	•	•		−
Pattyn 2011	CO	Belgium	52/70	51*/52*	35	•	40	60	•			•		•			•		•		•	•			•	+
Smolders 2011a	CO	Netherlands	92		39	•	35	65	•					•			•				•					−
Sunderman 1989	CO	USA	32				100			•				n. r.			•					•				−
Witzleb 2006	CO	Germany	185		49	•	40	60	•					•			•		•							−
Hart 2011a	CC	Great Britain	176		50		n. r.	n. r.	•						•		•	•	•	•		•				−
Bernstein 2011b	CS	Canada	34	60	50		59	41	•							•	•	•					•	•	•	−
Bisseling 2011	CS	Netherlands	57		40	•	63	37	•					•			•				•					−
Bolland 2011	CS	Great Britain	185	58	60		100		•						•		•		•			•	•	•		−
Braun 1986	CS	France	24				100		•						•											−
Brodner 2004	CS	Austria	60	62	55	•	100		•					n. r.			•						•		•	−
Campbell 2010	CS	USA	519					100	•								n. r.									−
Clarke 2003	CS	USA	44	57			50	50	•					•					•	•		•				−
Damie 2004	CS	France	48	70	38		100			•				•			•	•								−
Daniel 2006	CS	Great Britain	135	54*			38	62	•					•			•		•							−
Daniel 2008	CS	Great Britain	56	60*			100		•							•	•		•							−
Daniel 2010	CS	Great Britain	426	55			10	90	•							•	•		•	•						−
Davda 2011	CS	Great Britain	92	60	67		10	90	•					n. r.				•	•	•		•	•			−
De Haan 2008	CS	Belgium	214	51	42			100	•								•	•	•		•		•		•	−
De Smet 2008	CS	Belgium	26	53	73		23	77	•							•	•	•	•			•				−
Gleizes 1999	CS	France	41	54	51		100		•					•			•					•				−
Hallows 2011	CS	USA	46	57*	54		100		•	•						•						•	•	•	•	−
Hart 2006	CS	Great Britain	68	59*	43		50	50	•	•				n. r.			•	•	•			•				−
Hart 2008	CS	Great Britain	26	53	42	•		100	•										•				•			−
Hart 2009a	CS	Great Britain	26	52	65			100	•								n. r.						•	•		−
Hart 2009b	CS	Great Britain	139/164		34	•	35	65	•	•		•			•				•			•				−
Jacobs 1991	CS	USA	42	59*	45		100		•	•				n. r.			•									−
Karamat 2005	CS	Austria	50/75		46		100		•	•		•		•			•									−
Khan 2008	CS	Great Britain	21	54	38			100	•										•	•			•			−
Kwon 2010	CS	Great Britain	70	55*	56			100	•										•		•	•				−
Kwon 2011	CS	USA	178	56	34		11	89	•	•				•			•		•		•	•	•			−
Langton 2008+2009	CS	Great Britain	160	53*	40	•		100	•									•	•				•	•	•	−
Langton 2010	CS	Great Britain	247				8	92	•						•			•	•				•	•	•	−
Langton 2011a	CS	Great Britain	789					100	•									•	•		•		•	•	•	−
Langton 2011b	CS	Great Britain	723	53*	38			100	•									•	•		•		•	•	•	−
Langton 2011c	CS	Great Britain	257				20	80	•						•			•					•	•	•	−
Lhotka 2003	CS	Austria	259	55*	65		100		•					n. r.			•		•							−
Maclean 2010	CS	Great Britain	30					100	•								n. r.									−
Maezawa 2002	CS	Japan	75	67*	84		100		•	•				n. r.			•					•				−
Matthies 2011a	CS	Great Britain	120	57*	67		50	50	•						•		•	•	•	•		•	•	•		−
Matthies 2011b	CS	Great Britain	105		72		34	66	•						•		•	•	•	•		•	•	•		−
Migaud 2011	CS	France	30/62	40/40*	17	•	100		•				•	•			•					•				−
Milosev 2005	CS	Slovenia	43	56*	72	•	100		•					•			•					•				−
Moroni 2008	CS	Italy	46	48*	52		57	43	•					•			•		•							−
Moroni 2011	CS	Italy	95	54*	48	•	63	37	•					•			•		•				•			−
Pazzaglia 1983	CS	Italy	20	69			100			•				n. r.								•				−
Pelt 2011	CS	USA	39	56*	44		100		•						•							•	•	•	•	−
Pilger 2002	CS	Austria	46/53	58*/n. r.	54		100		•					n. r.					•							−
Rasquinha 2006	CS	USA	30/40	58*/58*			100		•	•			•	•			•					•				−
Saito 2006	CS	Japan	50/90	55*/n. r.	90		100		•	•				•			•							•		−
Sarmiento-González 2008	CS	Spain	22		46		100			•				n. r.								•				−
Savarino 2002	CS	Italy	41	54*	63		100		•	•				•			•									−
Savarino 2003	CS	Italy	41	49*	59		100		•					•			•									−
Savarino 2006	CS	Italy	42/65	57/59*	45		100		•			•		•			•					•				−
Schaffer 1999	CS	Austria	76	58*	61		100		•					n. r.					•							−
Tkaczyk 2010	CS	Canada	127	55*	63		100		•					•			•								•	−
Triclot 2009	CS	France	30/39			•	100		•					•			•									−
Underwood 2011	CS	Great Britain	130	56*	65			100	•									•	•				•	•		−
Walter 2008	CS	Australia	29					100	•										•							−
Williams 2011	CS	Canada	75			•	73	27	•	•						•	•						•		•	−
Akihiko 2011	CA	Japan	20	51			100		•					•			•									−
Allan 2007	CA	USA	35	51	43	•		100	•											•						−
Castelli 2011	CA	Italy	53				100		•						•			•								−
Corradi 2011	CA	Great Britain	31	62	23	•		100	•											•						−
Daniel 2007a+2009	CA	Great Britain	26	53		•		100	•										•				•		•	−
Daniel 2007b	CA	Great Britain	262	56	28		n. r.^$^	n. r.^$^	•							•	•		•	•						−
Delaunay 2000	CA	France	58	60	36	•	100		•					•			•									−
Delaunay 2004	CA	France	89	60	37		100		•					•			•							•		−
Desy 2011	CA	Canada	91	53	19			100	•									•					•	•	•	−
Girard 2011	CA	France	22	44	73	•	100		•					•			•						•			−
Grübl 2007	CA	Austria	22/98	n. r./56		•	100		•					•			•								•	−
Hart 2011b	CA	Great Britain	100		51	•		100	•								•	•	•	•		•	•	•		−
Imanishi 2010	CA	Japan	33	60	88	•	100		•						•			•					•			−
Isaac 2009b	CA	Great Britain	77		27			100	•									•								−
Kim 2011	CA	Canada	97	48	22	•		100	•												•		•	•	•	−
Ladon 2004	CA	Great Britain	95				100		•					•			•									−
Maezawa 2004	CA	Japan	44	63	80	•	100		•					•			•									−
Marker 2008	CA	Austria	70/98	n. r./56			100		•					•			•									-
Masse 2003	CA	Italy	30	52	67		100		•							•	•									−
Nikolaou 2011	CA	Canada	166	50	46		100		•							•	•	•					•	•	•	−
Skipor 2002	CA	USA	25	49	32			100	•												•					−
Vendittoli 2011	CA	Canada	29	50	48	•	100		•						•		•						•		•	−
Yang 2011	CA	China	25	37		•		100	•												•					−

Gathered information refer to patients with at least one metallic part of the bearing being investigated on metal ions.

Studies were summarized due to multiple reporting of identical patient population.

Study design:.

CO: Cohort study, examination on metal ions at 2 or more points of time, reference group being different regarding state of surgery, implant type, bearing.

or the like is necessary; incl. metal ion measurement for all groups.

CC: Case-control study, cases with elevated metal ion concentrations, controls with not elevated metal ions concentrations, retrospective detection of exposition (bearing, implant typ); incl. metal ion measurement for all groups.

CS: Cross-sectional study, examination on metal ions at one point of time, reference group being different regarding state of surgery, implant type, bearing or the like is necessary; incl. metal ion measurement for all groups.

CA: Case series, examination of 2 or more persons on metal ions, at one or more points of time; incl. metal ion measurement.

1: Number of patients (with minimum one metallic part of the bearing) being examined on metal ions/total number of patients being examined on metal ions.

2: Rounded mean age of patients with minimum one metallic part of the bearing/rounded mean age of total group of examined patients.

* : Rounded weighted mean.

3: BMI  =  Body Mass Index.

4: THA  =  Total hip arthoplasty.

5: HRA  =  Hip resurfacing arthroplasty.

MoM: Metal-Metal bearing.

MoP: Metal-Polyethylene bearing.

MoC: Metall-Ceramic bearing.

CoC: Ceramik-Ceramik bearing.

CoP: Ceramik-Polyethylene bearing.

n. r.: Not reported.

6: Femorale head size 28–32 mm; small head.

7: Femorale head size ≥36 mm; large head.

** Incl. Protek, AlloPro, Sulzer Orthop., Centerpulse, Zimmer.

*** Incl. Landander.

**** Incl. Midland med. Technologies, Medizintechnik Wien, Endo Plus.

UCLA: University of California, Los Angeles.

$ distribution was not reported for the whole group.

The vast majority of studies (n = 86; 83%) had significant methodological shortcomings such as a lack of reference group, lack of preoperative baseline assessment, lack of essential information on implant characteristics or metal ion measurement, and/or insufficient follow-up rates and were therefore considered as having a high risk of bias.

### Determination of metal ions in epidemiological studies

87 and 85 epidemiological studies reported Co and Cr values following hip replacement, respectively. No study differentiated between Cr(III) and Cr(VI). Metal ion concentration was most often measured in whole blood (n = 51), serum (n = 47), and urine (n = 19). Few studies investigated ion levels in erythrocytes (n = 2), plasma (n = 5), or in synovia (n = 3). Inductively coupled plasma mass spectrometry (ICP-MS) was used in 56 studies (60%), atomic absorption spectrometry (AAS) in 33 studies (36%), and inductively coupled plasma atomic emission spectrometry (ICP-AES) resp. inductively coupled plasma optical emission spectrometry (ICP-OES) in three studies for metal ion detection. Only 24studies (26%) reported preoperative (baseline) metal ion concentrations. For more details on metal ion assessment in the epidemiological studies included please refer to table 7.

**Table 7:** Points of time, investigated mediums, methods of analysis, and reporting of results of metal ion measurement in included epidemiological studies.

Reference	Metal ion measurement^1^					Metal ions		Medium				Technique		Outcome reported
	Preoperative	≤6 Months	>6–≤12 Months	>12––≤24 Months	>24 Months	Co	Cr	Whole blood	Erythrocytes	Serum	Urine^2^	AAS^3^	ICP-MS^4^	
Antoniou 2008		•	•			•	•	•					•	median, IQR
Beaulé 2011	•	•	•	•		•	•			•			•	mean, range, median, IQR
Bernstein 2011a		•	•			•	•	•					•	median, IQR
Boyer 2009	•	•	•	•	•					•				median, IQR
De Souza 2010	•	•	•	•	•	•	•			•		•	•	mean
Garbuz 2010	•	•	•	•		•	•			•			•	median, IQR
Isaac 2009a	•	•	•		•	•	•	•					•	median
Jacobs 1998	•		•		•	•	•			•	•	•		mean, range
Lavigne 2011	•	•	•	•		•	•	•					•	mean, range, median
Lazennec 2009	•	•	•	•	•	•	•			•		•		median, IQR
Pattyn 2011	•	•	•	•		•	•	•					•	mean, range, SD, median, IQR
Smolders 2011a	•	•	•	•		•	•	•		•			•	median, range, IQR
Sunderman 1989	•	•	•	•	•	•	•			•	•	•		mean, SEM, range
Witzleb 2006		•	•	•		•	•			•		•		median, IQR
Hart 2011a					•	•	•	•					•	median, range
Bernstein 2011b				•		•	•	•					•	median, IQR
Bisseling 2011	•		•			•	•	•		•			•	median, range
Bolland 2011					•	•	•	•					•	median, range
Braun 1986				•	•	•	•				•	•		mean, range
Brodner 2004					•	•	•			•		•		median
Campbell 2010	n. r.					•	•			•		n. r.		n. r.
Clarke 2003				•		•	•			•			•	median, SEM, range
Damie 2004					•	•	•	•					•	n. r.
Daniel 2006			•	•	•	•	•	•			•		•	mean
Daniel 2008			•	•	•	•	•	•			•	n. r.		mean
Daniel 2010	n. r.					•					•		•	median, IQR
Davda 2011					•	•	•	•					•	mean, range, median, IQR
De Haan 2008					•	•	•			•			•	mean, range, IQR
De Smet 2008					•	•	•			•			•	median, range, IQR
Gleizes 1999				•		•				•		•		mean, SD, range
Hallows 2011					•	•	•			•			•	median, range
Hart 2006				•	•	•	•	•					•	mean
Hart 2008					•	•	•	•					•	mean, SD
Hart 2009a	n. r.					•	•	•					•	median, range, IQR
Hart 2009b					•	•	•	•					•	median, IQR
Jacobs 1991					•					•	•	•		mean, range
Karamat 2005					•	•	•	•					•	median, range
Khan 2008					•	•							•	mean, range, SD
Kwon 2010					•	•	•			•			•	median, range, IQR
Kwon 2011					•	•	•			•			•	median, range, IQR
Langton 2008+2009					•	•	•	•		•			•	mean, range, median, IQR
Langton 2010					•	•	•	•		•			•	mean, range, median, IQR
Langton 2011a					•	•	•	•		•			•	median, range, IQR
Langton 2011b					•	•	•			•			•	median
Langton 2011c					•	•		•		•		n. r.		median
Lhotka 2003		•	•		•	•	•	•				•		mean, SEM
Maclean 2010				•		•	•	•			•	n. r.		n. r.
Maezawa 2002			•		•	•	•			•	•	•		mean, range
Matthies 2011a					•	•	•	•				n. r.		median, range
Matthies 2011b					•	•	•	•					•	mean, median, range
Migaud 2011					•	•	•	•					•	mean, SD, range
Milosev 2005	•	•	•	•	•	•	•			•		•		mean, SD, range, median, IQR
Moroni 2008				•	•	•	•			•		•		mean, SEM, median, range
Moroni 2011				•	•	•	•			•		•		mean, SD, range, median
Pazzaglia 1983					•		•	•			•	•		mean, SD
Pelt 2011					•	•	•			•			•	median, IQR, range
Pilger 2002			•	•	•	•	•	•			•	•		median, range
Rasquinha 2006					•	•	•			•		•		mean, SD, range, median
Saito 2006					•		•			•		•		mean, SD, range
Sarmiento-González 2008				•	•	•	•	•			•		•	mean, SEM, IQR
Savarino 2002				•	•	•	•			•		•		mean, SEM, median, range
Savarino 2003					•	•	•			•		•		mean, SEM, median, range
Savarino 2006					•	•	•			•		•		mean, SEM, median, range
Schaffer 1999			•	•	•	•	•	•			•	•		median, range, IQR
Tkaczyk 2010		•	•	•	•	•	•	•					•	median, IQR
Triclot 2009					•	•	•	•				•	•	mean
Underwood 2011					•	•	•	•					•	mean, range
Walter 2008		•	•	•	•	•	•	•	•	•		•	•	mean, median
Williams 2011				•	•	•	•			•			•	median, SD, range
Akihiko 2011			•	•	•		•			•		•		mean, range, median, IQR
Allan 2007	•	•	•	•	•	•	•			•			•	mean, SD, median
Castelli 2011	•	•	•	•	•	•	•	•					•	median
Corradi 2011					•	•	•	•			•		•	median, range, IQR
Daniel 2007a+2009	•	•	•	•	•	•	•	•			•		•	mean, range, median, IQR
Daniel 2007b		•	•	•	•	•	•	•		•			•	mean
Delaunay 2000					•	•		•				•		n. r.
Delaunay 2004			•		•	•		•				•		mean
Desy 2011					•	•	•	•					•	median
Girard 2011					•	•	•	•					•	mean, range, median
Grübl 2007					•	•	•			•		•		median, range
Hart 2011b					•	•	•	•					•	median, range, IQR
Imanishi 2010	•	•	•			•	•			•		•	•	median, IQR
Isaac 2009b	•	•	•	•		•	•	•					•	median
Kim 2011	•	•	•	•		•	•		•	•	•		•	mean, SD, range, median, IQR
Ladon 2004	•	•	•	•		•	•	•					•	median
Maezawa 2004		•	•	•	•		•			•		•		mean, SD, range
Marker 2008					•	•	•			•		•		median, range
Masse 2003	•	•				•	•	•			•	•		mean, SD, median
Nikolaou 2011	•		•	•	•	•	•	•					•	median, IQR
Skipor 2002	•	•	•			•	•			•	•	•		mean
Vendittoli 2011	•	•	•			•	•	•					•	mean, SD, range, median, IQR
Yang 2011	•	•	•	•		•	•			•	•	•		mean, SD, range

Studies were summarized due to multiple reporting of identical patient population.

2: Incl. all kinds of metal ion measurement in urin, e.g. 12- or 24 h urine.

3: AAS  =  All procedures of atomic absorption spectrometry.

4: ICP-MS  =  all procedures of inductively coupled plasma mass spectrometry.

n. r.  =  Not reported.

SD: Standard deviation; SEM: Standard error of the mean; IQR: Interquartile range.

### Metal ion concentrations in epidemiological studies

Table 8 summarizes median serum Co-concentrations, 75th percentiles, and maximum values of Co-concentrations before and after hip replacement stratified by the type of intervention (MoM SH-THA, MoM LH-THA, HRA). After MoM SH-THA median Co-concentrations varied between 0.65 and 1.5 µg/L at six months and between 0.7 and 1.7 µg/L two years postoperatively [42], [45], [46]. After HRA, median serum Co-concentrations varied between 1.12 and 3.7 µg/L six months and between 0.54 and 4.28 µg/L two years postoperatively indicating higher Co-levels after HRA vs. MoM SH-THA [45], [46], [47], [48], [49], [50]. Median Co-concentrations after MoM LH-THA varied between 0.7 and 3.26 µg/L six months and between 3.77 and 5.38 µg/L two years postoperatively [48], [49], [51].

**Table 8:** Median serum Co concentration in µg/L before and after hip replacement in epidemiological studies.

Serum Cobalt-values (µg/L) pre- vs. postoperative					
Intervention	Preoperative	≤6 Months	>6– ≤12 Months	>12– ≤24 Months	>24 Months
MoM SH-THA					
**range of medians**	**0.1**	**0.65–1.5**	**0.8–1.4**	**0.7–1.7**	**0.26–1.55**
number of studies	1	2	3	3	10
**range of 75th percentile**		**1,7**	**0.9–2,5**		**1.8**
number of studies		1	2		1
**range of maximum values**	**1.3**	**4.1**	**1.9**	**1.4**	**0.92–50.1**
number of studies	1	1	2	2	9

MoM LH-THA					
**range of medians**	**0.09–0.3**	**0.7–3.26**	**1.1–5.09**	**3.77–5.38**	**0.7–4.5**
number of studies	3	2	3	2	5
**range of 75th percentile**	**0.2 –0.3**	**0.9**	**1.65–7.5**	**7.2**	**1.15**
number of studies	2	1	2	1	1
**range of maximum values**	**0.57**	**10.25**	**12.85**	**9.59**	**5.6–79.3**
number of studies	1	1	1	1	4

HRA					
**range of medians**	**0.08–0.28**	**1.12–3.7**	**0.51–4.31**	**0.54–4.28**	**0.83–2.82**
number of studies	4	4	6	6	10
**range of 75th percentile**	**0.2**	**4,5**	**0.7–6**	**0.7**	
number of studies	1	1	3	1	.
**range of maximum values**	**0.19–2.6**	**5.09–11.4**	**7.42–11.4**	**7.13–17.6**	**8.96–228**
number of studies	3	2	3	4	7

One important result of our systematic review is that the maximum serum Co-levels were consistently higher at all postoperative assessments in patients who received MoM LH-THA [13], [48], [49], [51], [52], [53], [54], [55] and HRA [6], [7], [13], [45], [46], [47], [48], [49], [50], [53], [55], [56], [57], [58], [59], [60] compared to patients who received MoM SH-THA [45], [46], [50], [52], [56], [59], [60], [61], [62], [63], [64], [65], [66], [67] (Table 8).

Tables 9 and 10 summarize the results of comparative epidemiological studies. In accordance with these indirect comparisons from epidemiological studies, median Co-concentrations following MoM LH-THA and HRA tended to be higher compared to MoM SH-THA. Consistently, MoM LH-THA, MoM SH-THA, and HRA resulted in higher metal ion concentrations than THA with CoP, CoC, and MoP-implants.

**Table 9:** Co concentration in µg/L in serum/whole blood for MoM LH-THA or HRA vs. MoM SH-THA in epidemiological studies.

	Cobalt µg/L - Intervention (Median; IQR; n)						
Reference	Intervention	Medium	Preoperative	6 Months (±3)	12 Months (±3)	24 Months (±6)	48 Months (±6)
Antoniou 2008	MoM LH-THA	Whole blood		1.8; 58*	2.3; 58*		
	HRA	Whole blood		2.3; 70*	2.4; 70*		
Pattyn 2011	HRA_1	Whole blood	0.45; 22*	1.1; 22*	0.95; 0.73–1.2; 22	0.8; 0.6–0.9; 21	
	HRA_2	Whole blood	0.45; 20*	1.55; 20*	1.7; 1.1–2.4; 18	1.6; 1.2–2.1; 18	
Smolders 2011a	HRA	Serum	0.1; 0.1–2.6; 60**	1.2; 0.1–11.4; 51**	1.3; 0.1–11.4; 42**	1.5; 0.7–17.6; 21**	
Witzleb 2006	HRA	Serum		2.2; 56*	3.1; 50*	4.3; 23*	
Bisseling 2011	HRA	Serum	not reported		1.1; 0.1–7.7; 36**		
Clarke 2003	HRA	Serum				2.2; 22*	
Hallows 2011	MoM LH-THA	Serum					0.7; 0.0–14.0; 10** ^^2^^
Moroni 2011	HRA_1	Serum				0.55; 0.08–8.96; 15**	
	HRA_2	Serum					0.72; 0.3–5.6; 20** ^^3^^
Daniel 2006	HRA	Whole blood			1.3; 26***		
Daniel 2008	MoM LH-THA	Whole blood			2.3; 28***		
Garbuz 2010	MoM LH-THA	Serum	0.11; 0.1–0.2; 13		5.09; 3.0–7.5; 13	5.38; 3.5–7.2; 13	

	Cobalt µg/L – Comparison (Median; IQR; n)						
Reference	Comparison	Medium	Preoperative	6 Months (±3)	12 Months (±3)	24 Months (±6)	48 Months (±6)
Antoniou 2008	MoM SH-THA	Whole blood		2.5; 28*	2.6; 28*		
							
Pattyn 2011	MoM SH-THA	Whole blood	0.45; 10*	1.4; 10*	1.7; 0.7–2.2; 9	1.35; 0.9–2; 8	
							
Smolders 2011a	MoM SH-THA	Serum	0.1; 0.1–1.3; 32**	0.65; 0.1–4.1; 30**	0.8; 0.1–1.9; 23**	0.7; 0.1–1.4; 8**	
Witzleb 2006	MoM SH-THA	Serum		1.5; 18*	1.4; 13*	1.7; 34*	
Bisseling 2011	MoM SH-THA	Serum	not reported		0.8; 0.1–1.9; 21**		
Clarke 2003	MoM SH-THA	Serum				1.3; 22*	
Hallows 2011	MoM SH-THA	Serum					1.0; 0.3–14.0;10** ^2^
Moroni 2011	MoM SH-THA_1	Serum				0.98; 0.34–5.32; 25**	
	MoM SH-THA_2	Serum					0.86; 0.08–7.31; 35** ^3^
Daniel 2006	MoM SH-THA	Whole blood			1.7; 20***		
Daniel 2008	MoM SH-THA	Whole blood			1.7; 20***		
Garbuz 2010	HRA	Serum	0.13; 0.1–0.2; 13		0.51; 0.4–0.7; 13	0.54; 0.4–0.7; 13	

Values of the given studies were reported, if they fitted to the fixeds timeframes; if several measurements fitted to one timeframe, those values.

were reported being nearest to the timeframes given above; e. g. values were reported 3 months and 5 months postoperative > values 5 months.

postoperatively are shown in this table.

* Median; n.

** Median; range; n.

*** Mean; n.

^2^ metal ion measurement at mean 73 months postoperatively.

^3^ metal ion measurement mean 5 years postoperatively.

**Table 10:** Co concentration in serum/whole blood for MoM SH-THA, MoM LH-THA or HRA vs. MoP, CoP, CoC, MoC THA in epidemiological studies.

	Cobalt µg/L – Intervention (Median; IQ-Range; n)						
Reference	Intervention	Medium	Preoperative	6 Months (±3)	12 Months (±3)	24 Months (±6)	48 Months (±6)
Antoniou 2008	HRA	Whole blood		2.3; 70*	2.4; 70*		
	MoM LH-THA	Whole blood		1.8; 58*	2.3; 58*		
	MoM SH-THA	Whole blood		2.5; 28*	2.6; 28*		
Hart 2006	HRA	Whole blood				4.18; 34***	
							
Karamat 2005	MoM SH-THA	Whole blood				0.69: 0.19–3.7; 25**	
Savarino 2002	MoM SH-THA	Serum				0.97; 0.34–5.32; 26**	
Hallows 2011	MoM SH-THA	Serum					1.0; 0.3–14.0;10** 4
	MoM LH-THA	Serum					0.7; 0.0–14.0; 10** 4
Rasquinha 2006	MoM SH-THA	Serum					1.55; 0.58–7.93; 10** 4
							
Pattyn 2011	HRA_1	Whole blood	0.45; 22*	1.1; 22*	0.95; 0.73–1.2; 22	0.8; 0.6–0.9; 21	
	HRA_2	Whole blood	0.45; 20*	1.55; 20*	1.7; 1.1–2.4; 18	1.6; 1.2–2.1; 18	
	MoM SH-THA	Whole blood	0.45; 10*	1.4; 10*	1.7; 0.7–2.2; 9	1.35; 0.9–2; 8	
Hart 2009b	HRA	Whole blood					1.71; 1.29–2.33; 88
Isaac 2009	MoM SH-THA	Whole blood	0.45; 19*	0.51; 19*	0.83; 19*	1.0; 19* ^2^	

	Cobalt µg/L – Comparison (Median; IQ-Range; n)						
Reference	Comparison	Medium	Preoperative	6 Months (±3)	12 Months (±3)	24 Months (±6)	48 Months (±6)
Antoniou 2008	MoP THA	Whole blood		1.8; 18*	1.7; 18*		
							
							
Hart 2006	MoP THA	Whole blood					2.48; 34*** ^3^
							
Karamat 2005	MoP SH-THA	Whole blood				0.19: 0.19–1.07; 25**	
Savarino 2002	MoP SH-THA	Serum				0.62; 0.19–1.45; 15**	
Hallows 2011	MoP SH/LH-THA	Serum					0.35; 0.1–0.9; 10** ^3^
							
Rasquinha 2006	MoP SH-THA	Serum					0.26; 0.15–3.91; 10** 4
	CoP SH-THA	Serum					0.15; 0.15–0.40; 10** 4
Pattyn 2011	CoC SH-THA_1	Whole blood	0.6; 8*	0.5; 8*	0.5; 8*	0.45; 8*	
	CoC SH-THA_2	Whole blood	0.5; 10*	0.5; 10*	0.5; 10*	0.5; 10*	
							
Hart 2009b	CoC THA	Whole blood				0.21; 0.17–0.25; 25	
Isaac 2009	MoC SH-THA	Whole blood	0.55; 22*	0.5; 22*	0.72; 22 *	0.9; 22* ^2^	

* Median; n.

** Median; range; n.

*** Mean; n.

^2^ metal ion measurement at 34 months postoperatively.

^3^ metal ion measurement at 5 years postoperatively.

4 metal ion measurement at 36 months postoperatively.

Conclusions on the role of patient characteristics (age, sex) on metal ion concentration could not be drawn due to a lack of standardization in the design and reporting of the epidemiological studies included.

The levels of Cr and other metal ions showed similar distributions and lead to the same conclusions as the Co-ion levels reported (data available on request from the corresponding author).

### Course of metal ion concentration pre- vs. postoperative

**Figure 2:**
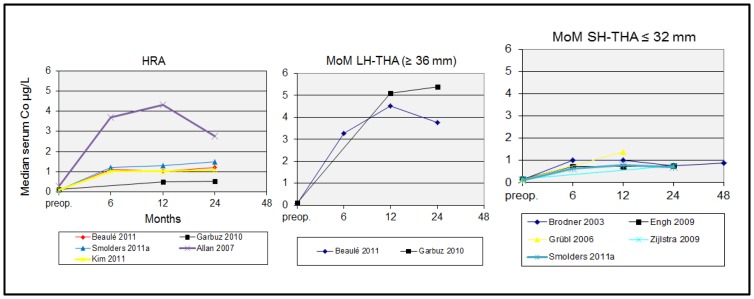
Course of median serum Co-concentration after implantation of hip replacements with different kinds of metal-on-metal bearings. Figure 2 provides an overview of the course of metal ion concentrations in studies reporting baseline serum Co-values and at least 2 postoperative Co-measurements. All MoM-interventions showed an increase in median serum Co-concentration. Again, highest median levels were observed in patients with HRA or MoM LH-THA. In some studies median Co-concentrations peaked at 12 months follow-up and declined thereafter. Other studies showed stable (increased) median serum Co-concentrations until 4-years follow-up.

Figure 2 provides an overview of the course of metal ion concentrations in studies reporting baseline serum Co-values and at least 2 postoperative Co-measurements. All MoM-interventions showed an increase in median serum Co-concentration. Again, highest median levels were observed in patients with HRA or MoM LH-THA. In some studies median Co-concentrations peaked at 12 months follow-up and declined thereafter. Other studies showed stable (increased) median serum Co-concentrations until 4-years follow-up.

### Maximum Co-concentrations in epidemiological studies

Authors applied different definitions of outlier/extreme values of Co-concentration ranging between 0.25 and 124.9 µg/L for all bearings. Details on the reported maximum Co-concentrations in epidemiological studies are provided in table 11. MoP THA generally resulted in lower extreme values than MoM hip replacements. In studies investigating MoM SH-THA maximum Co-concentrations ranged between 0.72 and 26.0 µg/L. The highest Co-concentrations were observed after MoM LH THA (range of maximum values: 1.8–79.3 µg/L) and after HRA (range of maximum values: 1.4–124.9).

**Table 11:** Maximal postoperative Co-concentrations after THA in RCTs and epidemiological studies.

Reference	Intervention	Outlier
**Brodner 2003**	not reported	overall postoperative: 2 outlier with Co-concentrations in serum of 24 and 119.2 µg/L
**Brodner 2004**	MoM THA	overall postoperative: 3 outlier with Co-concentrations in serum of 4.9–12.9 µg/L
**Dahlstrand 2009**	MoM SH-THA	overall postoperative: 7 outlier with Co-concentrations in serum up to >9 µg/L
	MoP THA	overall postoperative: 9 outlier with Co-concentrations in serum up to approx. 1.3 µg/L
**Engh 2009**	MoM LH-THA	overall postoperative: 2 outlier with Co-concentrations in serum of approx. 1.8 and approx. 3.4 µg/L
	MoM SH-THA	overall postoperative: 2 outlier with Co-concentrations in serum of approx. 2.0 and approx. 2.8 µg/L
	MoP THA	overall postoperative: 8 outlier with Co-concentrations in serum of approx. 0.25– approx. 2.75 µg/L
**Zijlstra 2009+2010**	MoM SH-THA	2 years postoperative: 2 outlier with Co-concentrations in serum of 7.0 and 15.6 µg/L
	not reported	5 years postoperative: 1 outlier with Co-concentration in serum of 7.0 µg/L
	not reported	10 years postoperative: 1 outlier with Co-concentrations in serum of 11 µg/L
**Allan 2007**	HRA	outlier: 1.5-fold boxwidth above 75th percentile
		overall postoperative: 6 outlier Co-concentrations in serum of 13.6–124.9 µg/L
**Antoniou 2008**	MoM SH-THA	overall postoperative: 5 outlier with Co-concentrations in whole blood of approx. 4– approx. 6.5 µg/L
	MoM LH-THA	overall postoperative: 9 outlier with Co-concentrations in whole blood of approx. 2.5– approx. 10 µg/L
	HRA	overall postoperative: approx. 11 outlier with Co-concentrations in whole blood of approx. 3.3–approx. 11.7 µg/L
**Bernstein 2011a**	MoM SH-THA	overall postoperative: 6 outlier with Co-concentrations in whole blood of approx. 2.5– approx. 19 µg/L
	MoM LH-THA	overall postoperative: 3 outlier with Co-concentrations in whole blood of approx. 24.0– approx. 37.5 µg/L
**Bernstein 2011b**	MoM SH-THA + MoM LH-THA + HRA	whole cohort consists of oulier with Co-concentrations in whole blood ≥10 µg/L
**Daniel 2010**		outlier: values above upper quartile + 3-fold IQ-range
	not reported	overall postoperative: 14 outlier with Co-concentrations in plasma of approx. 8.8– approx. 14.5 µg/L
**De Haan 2008**	HRA (steep)	overall postoperative: 9 outlier with Co-concentrations in serum of approx. 10– approx. 112 µg/L
	HRA (non-steep)	overall postoperative: approx. 12 outlier with Co-concentrations in serum of approx. 5– approx. 30 µg/L
**Delauanay 2000**	MoM SH-THA	overall postoperative: 6 patients with Co-concentrations in whole blood >5 µg/L
**Delaunay 2004**	MoM SH-THA	laboratory reference value value 5 µg/L for Co in whole blood
		overall postoperative: 15 outlier with Co-concentrations in whole blood >5 µg/L with max. 36 µg/L

Reference	Intervention	Outlier
**De Smet 2008**	MoM (unilateral)	overall postoperative: 1 outlier with Co-concentration in serum of approx. 93 µg/L
	MoM (bilateral)	overall postoperative: 1 outlier with Co-concentration in serum of apptox. 62 µg/L
**Garbuz 2010**	HRA	overall postoperative: 2 outlier with Co-concentrations in serum of approx. 1.5+1.8 µg/L
	MoM LH-THA	overall postoperative: 1 outlier with Co-concentrations in serum of approx. 2.4 µg/L
**Grübl 2007**	MoM SH-THA	overall postoperative: 2 outlier with Co-concentrations in serum 8.4 resp. 50.1 µg/L
**Hallows 2011**	MoM SH-THA	overall postoperative: 1 outlier with Co-concentration in serum of 14.0 µg/L
	MoM LH-THA	overall postoperative: 1 outlier with Co-concentration in whole blood of 79.3 µg/L
**Imanishi 2010**	MoM LH-THA	overall postoperative: 5 outlier with Co-concentrations in serum approx. 2.2– approx. 4.6 µg/L
**Isaac 2009a**	MoM SH-THA + CoC SH-THA	overall postoperative: 8 outlier with Co or Cr-concentrations in whole blood > 0 µg/L
**Isaac 2009b**	HRA	overall postoperative: 6 outlier with Co-concentrations in whole blood >10 µg/L
**Kim 2011**	HRA	overall postoperative: approx. 14 outlier with Co-concentrations in serum approx. 3.0– approx. 49 µg/L
**Kwon 2010**	HRA (no pseudotumor)	overall postoperative: 3 outlier with Co-concentrations in serum approx. 7.0– approx. 9.5 µg/L
**Kwon 2011**	MoP THA	overall postoperative: 1 outlier with Co-concentrations in serum of 3.8 µg/L
	HRA (no pseudotumor)	overall postoperative: 4 outlier with Co-concentrations in serum approx. 6.0– approx. 10.5 µg/L
**Langton 2011a**	HRA (incl. ASR)	overall postoperative: several outlier with Co-concentrations in serum of approx. 20 µg/L–240 µg/L
**Langton 2011b**	HRA – DePuy (ASR)	overall postoperative: 56 outlier (23 %) with Co-concentrations in serum >7 µg/L
	HRA – Smith&Nephew/Wright Medical Technology	overall postoperative: 29 outlier (6 %) with Co-concentrations in serum >7 µg/L
**Langton 2011c**	HRA – DePuy (ASR); MoM LH-THA – DePuy	overall postoperative: 67 outlier (26,1 %) with Co- or Cr-concentrations in serum >7 µg/L
	HRA – DePuy (ASR); MoM LH-THA – DePuy	overall postoperative: 30 outlier (11,7 %) with Co- or Cr-concentrations in serum >20 µg/L
**Lavigne 2011**	MoM LH-THA – Zimmer	overall postoperative: 2 outlier with Co-concentrations in whole blood of approx. 6.3 and approx. 6.8 µg/L
	MoM LH-THA – Biomet	overall postoperative: 14 outlier with Co-concentrations in whole blood of approx. 1.5– approx. 13.0 µg/L
	MoM LH-THA – DePuy	overall postoperative: 9 outlier with Co-concentrations in whole blood of approx. 2.2–approx. 8.4 µg/L
	MoM LH-THA – Smith&Nephew	overall postoperative: 5 outlier with Co-concentrations in whole blood of approx. 3.0– approx. 5.2 µg/L
**Lazennec 2009**	MoM SH-THA	overall postoperative: 1 outlier with highest Co-concentration in serum of 47 µg/L

Reference	Intervention	Outlier
**MacDonald 2003**	MoM SH-THA	overall postoperative: 3 outlier with Co-concentrations in erythrocytes of approx. 7.0– approx. 26.0 µg/L
	MoP THA	overall postoperative: 1 outlier with Co-concentration in erythrocytes of approx. 3.0 µg/L
**Marker 2008**	MoM SH-THA	overall postoperative: 2 outlier with Co-concentrations in serum 50.1 and 60.6 µg/L
**Matthies 2011b**	not reported	more than half of the patients with pseudotumors with Co- or Cr-concentrations in whole blood > 7 µg/L
**Nikolaou 2011**	MoM LH-THA + MoM SH-THA	overall postoperative: 10 outlier with Co-concentrations in whole blood approx. 3.0– approx. 5.5 µg/L
**Pattyn 2011**	MoM SH-THA – Zimmer	overall postoperative: 2 outlier with Co-concentrations in whole blood of approx. 5.2 µg/L
	HRA – Zimmer	overall postoperative: 2 outlier with Co-concentrations in whole blood of approx. 1.4 and approx. 3.4 µg/L
	HRA – Smith&Nephew	overall postoperative: 3 outlier with Co-concentrations in whole blood of approx. 4.0– approx. 6.7 µg/L
**Pelt 2011**	MoM LH-THA	overall postoperative: 4 outlier (unilateral) with Co-concentrations in serum of approx. 1.2– approx. 5.6 µg/L
	MoM LH-THA	overall postoperative: 2 excluded outlier with Co-concentrations in serum of 5.6 (bilateral) +79.3 (unilateral) µg/L
**Pilger 2002**	MoM THA	overall postoperative: 16 outlier with Co-concentration in whole blood of >5 µg/L
**Savarino 2002**		reference value for Co of 0.72 µg/L (Italian health agency)
	MoM SH-THA	overall postoperative: 17 outlier (65 %) with Co-concentrations in serum > reference value
	MoP THA	overall postoperative: 90% with Co-concentrations in serum > reference limit 0.4 µg/L
**Savarino 2003**	MoM SH-THA	overall postoperative: 24 outlier with Co-concentrations in serum > median reference value of 0,29 µg/L for Co
**Savarino 2006**	MoM SH-THA	overall postoperative: 5 outlier (33 %) with Co-concentrations in serum > reference value of 0.4 µg/L for Co
**Schaffer 1999**	MoM THA	overall postoperative: 12 outlier with Co-concentration in whole blood of >5– approx. 33.5 µg/L
**Smolders 2011b**	MoM SH-THA	overall postoperative: 5 outlier with Co-concentration in whole blood of approx. 2.1– approx. 4.2 µg/L
	HRA	overall postoperative: 10 outlier with Co-concentration in whole blood of approx. 3.0–22.8 µg/L
**Tkaczyk 2010**	MoM SH-THA	overall postoperative: 12 outlier with Co-concentrations in whole blood of approx. 4.0– approx. 8.2 µg/L
**Vendittoli 2007+2010**		outlier: 1.5–3 boxwidth above 3. quartile; extreme values: >3 boxwidth above 3. quartile
	MoM SH-THA	overall postoperative: 5 outlier with Co-concentration in whole blood of approx. 2.5– approx. 5.7 µg/L
	HRA	overall postoperative: 9 outlier with Co-concentration in whole blood of approx. 3.0– approx. 6.5 µg/L
**Vendittoli 2011**	MoM LH-THA	overall postoperative: 2 outlier with Co-concentrations in whole blood approx. 5.8– approx. 7.0 µg/L
**Williams 2011**	MoM LH-THA	overall postoperative: 1 outlier with Co-concentration in serum >50µg/L
	HRA	overall postoperative: 2 outlier with Co-concentrations in serum of approx. 3.0 and >50 µg/L;
**Witzleb 2006**	MoM SH-THA	overall postoperative: 4 outlier with Co-Concentrations in serum approx. 5.5– approx. 9.5 µg/L
	HRA	overall postoperative: 4 outlier with Co-Concentrations in serum approx. 6.5– approx. 23 µg/L

Due to substantial differences in the design, interventions, methods of metal ion assessment, study populations and study reporting, we considered statistical meta-analysis not to be indicated.

### Local clinical reactions

**Figure 3:**
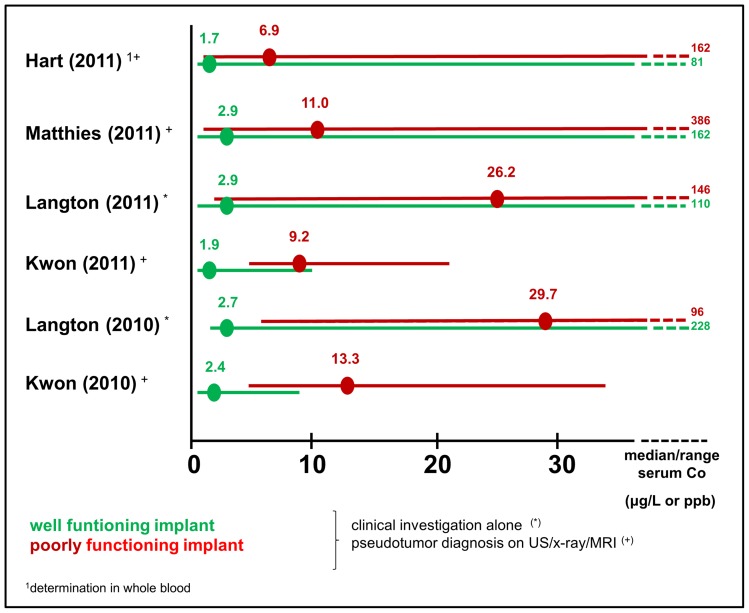
Summarizes six studies which reported Co-concentrations in well and poorly functioning implants [55], [18], [19], [20], [53]. Cases with local metal-related adverse reactions (poorly functioning implants) had consistently higher metal ion concentrations than patients with well-functioning THA.

Local metal-related adverse reactions were reported in 9 epidemiological studies [5], [6], [13], [19], [20], [53], [55], [68], [69]. As summarized in table 12 ARMD, metallosis and pseudotumors were the most frequently reported metal-related adverse reactions. Six studies reported Co-concentrations in well and poorly functioning implants [5], [18], [19], [20], [53]. Cases with local metal-related adverse reactions (poorly functioning implants) had consistently higher metal ion concentrations than patients with well-functioning THA (Figure 3).

**Table 12:** Documentation of local metal-related adverse reactions.

Reference	local clinical reactions
Bolland 2011	14 patients (7.6 %) with revision due to ARMD
DeSmet 2008	10 patients (38.5 %) with metallosis
Kwon 2010	10 patients (14.3 %) with pseudotumors diagnosed by MRI
Kwon 2011	7 patients (4 %) with pseudotumors diagnosed by MRI
Langton 2010	16 patients with ASR, revision due to ARMD
Langton 2011a	60 failures related to ARMD, incl. patients with ASR
Langton 2011c	82 failures (31.9 %) related to ARMD, patients with ASR HRA and ASR THA
Matthies 2011b	72 patients (68.6 %, incl. patients with ASR) with pseudotumors diagnosed by MARS-MRI
Williams 2011	15 patients (20 %) with pseudotumors diagnosed by ultrasound

ARMD  =  adverse reaction to metal debris.

ASR  =  Articular Surface Replacement, Firma DePuy.

MRI  =  Magnetic Resonance Imaging.

MARS-MRI  =  Metal Artifact Reduction Sequence-Magnetic Resonance Imaging.

### Systemic clinical reactions

Five studies [61], [62], [70], [71], [72] examined possible associations between metal ion concentrations and nephrotoxicity. Daniel et al. [71] examined renal clearance and renal concentrating efficiency of cobalt. The renal efficiency, i.e. the ratio of urine cobalt concentration to plasma cobalt concentration, was 0.9 (IQR 0.7–1.6) for preoperative controls and 3.2 (IQR 1.7–5.1) in patients with MoM THA or HRA. No threshold was endorsed at which renal capacity is overextended. Corradi et al. [70] examined metal ion concentrations in whole blood and renal markers in patients with HRA and in healthy controls. The median Co-excretion in patients with HRA was 12.9 µg/24-h urine (range 6.1–71.5 µg). No elevated renal markers were found in comparison with controls. Gruebl et al. and Marker et al. [61], [62] investigated serum metal ions, blood urea nitrogen, and serum creatinine in overlapping cohorts of patients with MoM THA. The median (range) serum creatinine value preoperatively and at 10 years follow-up was 0.88 mg/dL (0.63–1.21 mg/dL) and 0.86 mg/dL (0.55–1.51 mg/dL), respectively. Evidence for or against further systemic toxicity or carcinogenicity could not be revealed from the studies included.

## Discussion

As highlighted in this comprehensive systematic review, there is substantial and consistent evidence that patients receiving hip replacement with a MoM-bearing are at increased risk for systemic accumulation of metallic products. In the 104 studies analyzed, median metal ion concentrations were persistently elevated after implantation of MoM-bearings in all investigated mediums (whole blood, serum, plasma, erythrocytes, urine), irrespective of patient characteristics and study characteristics.

Overall, metal ion concentrations in body fluids were assessed in 9.957 patients in the 11 RCTs and 93 epidemiological studies included in this review. Despite heterogeneity in the study designs, techniques and medium of metal ion assessment, investigators consistently observed elevated median/mean metal ion concentrations after MoM THA and HRA compared to baseline, i.e. before surgery. Metal-free hip replacements did not result in increased metal ion levels. Metal ion concentrations following MoP and MoC THA were much lower compared to MoM THA or HRA.

One important finding from this review is that stemmed large-head MoM-implants and HRA tended to result in higher Co-concentrations than small-head MoM-implants. In several studies very high serum cobalt concentrations above 50 µg/L were measured in patients who had received large-head MoM-implants or HRA.

These findings have significant clinical relevance, as increased metal ion concentrations translate into increased risk for the development of local adverse reactions such as ARMD. In many cases ARMD results in the indication for the revision of MoM-implants.[13] One current issue of debate is the definition of a cutoff cobalt level, above which revision should be considered.

Hart et al. [8], [18], [30] recommend a serum cobalt threshold level of 4.97 µg/L based on ROC-curve analyses. However, no explicit advice is given on how to treat patients above this value. Recommendations of present literature currently state Co-concentrations in serum or plasma greater 2 up to 7 µg/L as a predictor for a subjectively adverse outcome and an increased risk of MoM-specific complications [8], [18], [30], [73].

While MoM SH-THA (head diameter ≤32 mm) seem to show similar long-term survival rates as hip replacement with other bearings [61], [74], [75], [76], [77], the implantation of stemmed LH-THA (head diameter ≥36 mm) is associated with significantly higher short-term revision rates in clinical studies as well as arthroplasty registries [15], [16], [17], [44], [45], [48], [68], [78], [79]. The elevated release of metal products in these stemmed LH-implants may be due to fretting corrosion at the head-taper-junction in addition to a metal particle release from bearing surfaces.

Beside local tissue damages, it is important to gain better understanding about the potential systemic adverse effects induced by metal ion accumulation, i.e. toxicity, carcinogenicity, teratogenicity. The degree to which increased metal ion concentrations after MoM THA translate into increased risk for systemic toxicity cannot be sufficiently answered based on the studies identified and analyzed in this review. Until now, epidemiological studies have not revealed clinically relevant toxic damages of the kidney, heart or nervous system after MoM THA [45], [58]-[60]. Case reports, however, indicate the possibility of metal-induced cardiomyopathy [21], [22]. An elevated risk of incident cancer following hip replacements with MoM bearing could not be identified yet [80], [81], [82], but studies may have been underpowered.

There is substantial evidence that occupational metal exposure is related to increased cancer risk. It has to be noted, however, that bioavailability of Cr(III) compounds is substantially lower than those of Cr(VI) compounds. Cr(VI) compounds are able to infiltrate into cells due to transmembrane motion and to operate genotoxic following reduction to Cr(III). Carbid metal workers exposed to Co are at increased risk for fatal lung cancer [83]; the „International Agency for Research on Cancer (IARC)“ classified Co to be possibly carcinogenic. Persons being occupationally exposed to Co have higher Co urine concentrations when compared to the general population. It should be noted that specific attentiveness was laid on possibly elevated Cr(VI) in body fluids due to ascertained carcinogenicity of Cr(VI) compounds. Due to considerable differences in exposure routes, the effects of increased metal ion concentrations as a consequence of MoM hip replacement cannot be directly compared with the systemic effects of occupationally acquired (mainly inhaled) metals.

### Strengths and weaknesses of this review

This systematic review was conducted in accordance with the PRISMA checklist [31]. Systematic literature search and assessment of eligibility of studies identified was done independently by two reviewers. Study quality assessment was based on a priori defined criteria. Due to methodological limitations in most of the studies included and due to substantial qualitative differences in the study design, conduct, and reporting, quantitative meta-analysis was not indicated. However, as highlighted above, the qualitative results are consistent despite the heterogeneity of the studies included so that we consider the conclusions drawn to be robust and generalizable.

### Implications for clinical practice and future research

After hip replacement with contemporary MoM bearings the release of metal ions is highest in stemmed implants with large heads followed by resurfacing devices and also – but on a lower level – small heads. As the deposition of metal products may not only lead to local but possibly also systemic adverse health outcomes, the conclusions of this review have high relevance not only for orthopaedic surgeons, but also for other medical disciplines.

Due to the risk of systemic accumulation of metal ions following implantation of hip replacements with MoM bearing, consideration on risks and benefits should be done carefully and individually for every patient prior to surgery.

The authors support a „time out“ of stemmed large-head MoM-THA and recommend a restricted indication for hip resurfacing arthroplasty to patients without risk factors such as small implant size, female gender, and renal insufficiency [5], [79]. Patients with status post implantation of MoM should be followed by standardized monitoring. Especially examined ions, medium and analysis technique should be standardized to allow comparability of results and further analysis. Close interdisciplinary cooperation is necessary in case of potential systemic reactions due to increased metal ion concentrations.

An approach to this unresolved difficulty was one of the main objectives of an international and interdisciplinary expert conference, which took place in our institution. In April 2012, we hosted an international multi-disciplinary expert conference endorsed by the “European Federation of National Associations of Orthopaedics and Traumatology” (EFORT), “European Hip Society” (EHS), and the “German Osteoarthritis Society” in order to provide clinically-relevant advice on how to treat and monitor current and future patients with MoM THR. Beside orthopaedic surgeons being experienced with MoM hip endoprosthetics, epidemiologists, toxicologists, biomechanics, and pathologists as well as a patients representative and regulatory agency representative from 7 European countries and the US participated.

The statement resulting from this consensus initiative is published in detail on web sites of European [30] and German [84], [85] orthopaedic societies [86]. Beside detailed recommendations on monitoring of MoM hip replacements and metal ion measurement the statement also summarizes prioritized questions for future research. One research issue that needs to be prioritized is the investigation of potential systemic risks due to accumulation of metal ions.
